# Design and Synthesis of Luminescent Lanthanide-Based Bimodal Nanoprobes for Dual Magnetic Resonance (MR) and Optical Imaging

**DOI:** 10.3390/nano11020354

**Published:** 2021-02-01

**Authors:** Walid Mnasri, Mahsa Parvizian, Souad Ammar-Merah

**Affiliations:** ITODYS Universite de Paris, CNRS UMR-7086, 15 Rue Jean-Antoine de Baîf, 75025 Paris, France; walid.tevez@hotmail.fr (W.M.); parvizian.mahsa21@gmail.com (M.P.)

**Keywords:** multimodal nanoprobes, MRI contrast agents, luminescent dyes, lanthanides, down-conversion, up-conversion, colloidal chemistry

## Abstract

Current biomedical imaging techniques are crucial for the diagnosis of various diseases. Each imaging technique uses specific probes that, although each one has its own merits, do not encompass all the functionalities required for comprehensive imaging (sensitivity, non-invasiveness, etc.). Bimodal imaging methods are therefore rapidly becoming an important topic in advanced healthcare. This bimodality can be achieved by successive image acquisitions involving different and independent probes, one for each mode, with the risk of artifacts. It can be also achieved simultaneously by using a single probe combining a complete set of physical and chemical characteristics, in order to record complementary views of the same biological object at the same time. In this scenario, and focusing on bimodal magnetic resonance imaging (MRI) and optical imaging (OI), probes can be engineered by the attachment, more or less covalently, of a contrast agent (CA) to an organic or inorganic dye, or by designing single objects containing both the optical emitter and MRI-active dipole. If in the first type of system, there is frequent concern that at some point the dye may dissociate from the magnetic dipole, it may not in the second type. This review aims to present a summary of current activity relating to this kind of dual probes, with a special emphasis on lanthanide-based luminescent nano-objects.

## 1. Introduction

Current biomedical imaging techniques are vital for the diagnosis of various diseases. Each imaging mode has its own merits and disadvantages and uses specific probes exhibiting particular physical and chemical properties. However, a single technique does not encompass all the functionalities required for comprehensive imaging. For instance, magnetic resonance imaging (MRI) has the advantage of being a non-invasive technique for in vivo 3D-tomography, but it is limited by low target sensitivity. Also, optical imaging (OI) is non-invasive and has the advantage of a high sensitivity and a high specificity, but it is limited by the poor light tissue penetration. Most of the time, it requires appropriate and expensive endoscopic optical fibers for local photoexcitation and/or detection.

Therefore, multimodal imaging methods, with enhanced signal sensitivity, better spatial resolution, and the ability to relay information about biological systems at the molecular and cellular levels are becoming important tools with an absolute necessity of designing multimodal probes combining ideally, in single objects, all the desired properties.

Nanoparticles (NPs) as platforms bringing together several functionalities offer such an opportunity. They can be easily produced by colloidal chemistry with different sizes allowing thus a strict control of their size-dependent physico-chemical properties (surface plasmon, quantum confinement, superparamagnetism) and then a strict control of their functionality. This functionality can be also tuned by replacing these NPs with their hetero-nanostructured counterparts bringing complementary abilities.

In MRI applications, magnetic structures, here called ‘contrast agents’ (CAs), accelerate the relaxation of water molecules by locally shortening the longitudinal T_1_ and transversal T_2_ relaxation times of the nuclear magnetic moment of their ^1^H protons [1]. In practice, paramagnetic species, mainly 3d- and 4f-block metal complexes [2], commonly called transition metal and lanthanide complexes, are suitable for positive contrast (MR images in which areas with low T_1_ are bright) while superparamagnetic ones, mainly Fe_3_O_4_ and γ-Fe_2_O_3_ NPs [3], are suitable for negative contrast (MR images in which areas of low T_2_ are dark). These particles can serve as a core platform for the addition of other functional moieties like fluorescent tags [4], radionuclides [5] or targeting biomolecules [6,7] to design bimodal MRI and OI probes.

In OI applications, light nanosources—commonly called biolabels—allow illuminating cellular and sub-cellular details. These sources are generally organic chromophores [8,9] luminescent lanthanide (Ln) chelates [10,11] or intrinsically optically active inorganic nanocrystals like metallic plasmonic particles (PPs) [12,13], semiconducting quantum dots (QDs) [14,15], including carbon dots (CDs) [16], and Ln doped up or (down) conversion ceramic nanoparticles, very often abbreviated as UCNPs and DCNPs [17]. All these dyes can be combined in a single architecture with para- or superparamagnets to form bimodal probes for both cancer cells MRI detection and subcellular fluorescence imaging. This was for instance achieved by embedding organic chromophores in silica to form a luminescent core on which magnetic iron oxide nanosatellites were attached [18] as well by coating UCNPs or DCNPs with a silica thin layer embedding paramagnetic Gd complexes [19].

Focusing on these two functionalities—magnetic and optical ones—several efforts have been carried out to build smart bimodal platforms, where simultaneous MR and optical imaging are desired. The aim of this review is to highlight the different strategies to engineer and prepare such dual probes and particularly those architected around luminescent Ln-based dyes. The choice of these systems instead of others is motivated by the exceptional biological, chemical, and physical properties of Ln light sources and particularly those of UCNPs. UCNPs, in contrast to all the other dyes, operate through the well-known up-conversion phenomenon, which is based on the absorption of lower-energy photons by lanthanide centers located in the crystal lattice of an inert matrix, mainly fluoride, oxyfluoride, or phosphate [17], and the emission of higher-energy photons thanks to efficient internal energy transfers. In relation to their chemical composition, they exhibit improved stability against photobleaching, photoblinking, and photochemical degradation, while they operate at low energy excitation light, in the biological optical transparency window, meaning their use without requiring any local photoexcitation instruments.

This review is thus organized into three main sections, a first one summarizing MRI principle and remembering CA classification, a second highlighting the requirement for an efficient OI and listing the existing Ln-based dyes, the molecular and the solid-state ones, and finally, a third section specifically dedicated to the description of the bimodal MRI and OI probes, constructed around these Ln emitting centers, with a special emphasis on their chemical processing strategies.

## 2. MR Imaging and Contrast Agents

MR imaging is based on the magnetic nuclear resonance of water protons in the body. With MRI, three-dimensional images of entire live specimens can be obtained with high resolution and without the use of ionizing radiation. The MR contrast is essentially derived from the environment variation of water protons leading to different signal intensities.

In practice, when a living body is placed in a large static magnetic field B_0_, many of the free hydrogen nuclear magnetic moments align themselves with the direction of the magnetic field. When a magnetic radio-frequency pulse B_RF_, with an appropriate frequency (the gyromagnetic resonance frequency) is applied perpendicular to B_0_, the nuclear magnetic moments of the hydrogen atoms tilt away from B_0_. At the end of the pulse, the magnetic moments are in the plane perpendicular to B_0_. They then relax to realign themselves parallel to B_0_, by reducing their B_RF_ induced transverse magnetization component (M_xy_) to zero and increasing their longitudinal magnetization component (M_z_) to its initial value, with characteristic T_2_ and T_1_ relaxation times, respectively.

The Bloch equations for the relaxation of the longitudinal (M_z_) and transverse (M_xy_) components of the nuclear magnetization of water protons to their equilibrium state indicate that the MRI signal is proportional to the proton density of the targeted tissues and to the product (1−*e*^−T_R_/T_1_^)^−T_E_/T_2_^, where T_R_ is the time of the B_RF_ pulse and T_E_ the time between the application of the B_RF_ pulse and the measurement of the response signal. In the presence of CAs, the T_1_ and T_2_ relaxation times are significantly shortened, affecting thus the MRI signal, inducing the desired water proton response contrasting.

The efficiency of CAs is usually expressed by the enhancement of the relaxation rate, also called relaxivity, r_i_, defined according to the following equation:

(1/T_i_) = (1/T_i_)_0_ + r_i_[CA] (1)

where (1/T_i_) is the inverse of T_i(=1 or 2)_ in the presence of CA, at a [CA] concentration, while (1/T_i_)_0_ is the inverse of T_i(=1 or 2)_ in its absence. It is generally stated in MRI textbooks that the ratio r_2_/r_1_ determines whether a given CA is more suitable as a T_1_ (positive) or as a T_2_ (negative) MRI agent. When CAs affect longitudinal and transverse relaxivities to a similar degree they are better suited for T_1_-weighted imaging and are commonly called positive CAs, whereas when they preferentially increase transverse relaxivity, typically r_2_/r_1_ >>1, they are better suited for T_2_-weighted imaging and are called negative CAs.

**Figure 1:**
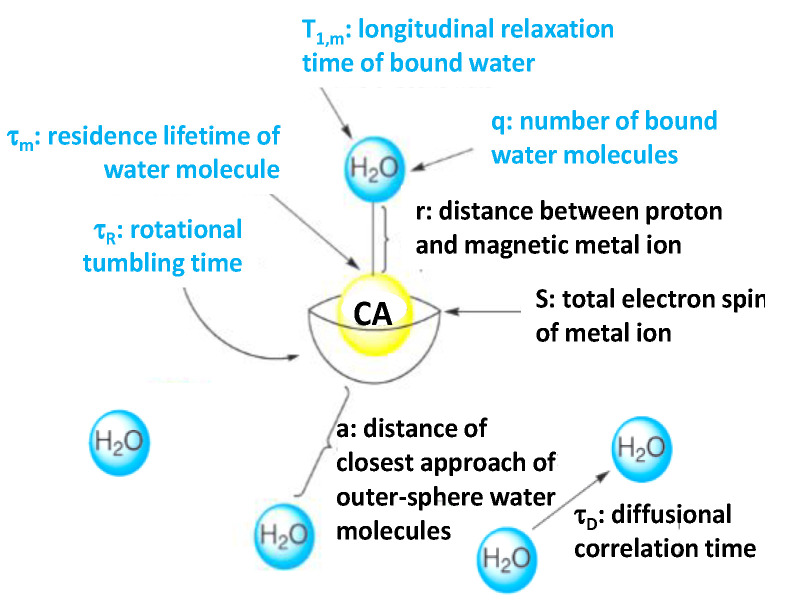
Variables contributing to CA relaxivity in the inner- (**in blue**) and outer-sphere (**in black**) mechanisms. Reproduced from [20], with permission from RSC, 2009.

The interaction between CAs and water protons is of two types, depending on whether the water molecule is directly attached to the CA or not. It is of inner-sphere type if it proceeds through a direct water attachment and it is of outer-sphere type if it does not (Figure 1), the former being more effective for positive CAs while the latter for the negative ones.

The Solomon–Bloembergen–Morgan equations are commonly used to describe the relationship between variables contributing to the inner-sphere interaction and the relaxivity [21,22]. They define the number of water molecules (q) directly attached to the CA, the residence lifetime of inner-sphere water molecules (τ_m_), and the rotational tumbling time (τ_R_) of the CA. Greater inner-sphere water access (i.e., higher q values) leads to larger relaxivity values. A similar trend is true for τ_R_, longer tumbling times yielding higher relaxivity values. The relationship between τ_m_ and relaxivity is more subtle. Nevertheless, it is accepted now that if τ_m_ is too small, the interaction between a water molecule and the CA is too short for the full relaxivity potential to be achieved [2]. In practice, the relaxivity is more often limited by slow exchange (due to a long τ_m_) when only a limited number of water molecules can be relaxed.

In the outer-sphere model introduced by Ayant et al. [23] and Freed et al. [24] for paramagnetic agents, and adapted by Gillis et al. to superparamagnetic ones [25], the increase in the relaxation rate 1/T_2_ compared to pure water originates from fluctuating dipolar interactions between nuclear spins of water protons and the electronic magnetic moment of the CAs. For a limited range of diameters called the ‘motional averaging regime’, the CA can be considered immobile during T_E_ compared to the random trajectories of water molecules diffusing around the CA sphere. In this case, Vuong et al. have shown that r_2_ follows a universal scaling law that is quadratic both with the magnetization and with *a*, the radius of the outer sphere also called the relaxometric size, defined as the minimum approach distance between H_2_O molecules and the CA center [26]. The diffusional correlation time of outer-sphere water molecules (τ_D_) can also contribute to the CA relaxivity, even if the relationship between τ_D_ and r_2_ is not evident.

Finally, CAs must be non-toxic and they must be engineered accordingly. An agent with a high relaxivity value allows reducing its dose and, thus, its potential toxicological effect. These two criteria have to be absolutely respected to allow CA clinical use and consequently to drive their design and synthesis [2].

### 2.1. Positive Contrast Agents

**Figure 2:**
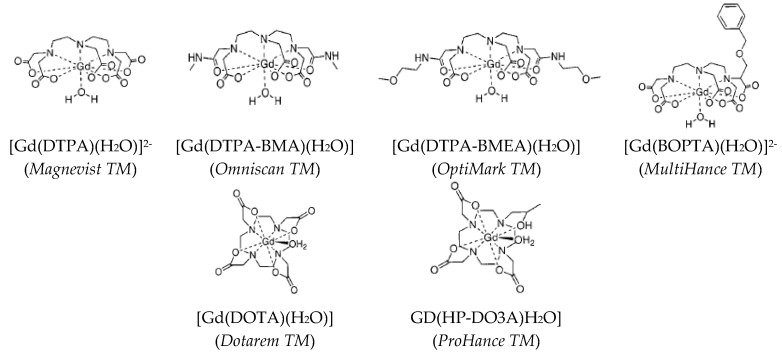
The semi-developed formula of Gd-chelates involving linear (**top**) or cyclic (**down**) ligands (the commercial names are given in parenthesis). Reproduced from [2], with permission from ACS, 1999

Positive CAs are mainly (Figure 2) paramagnetic metallic complexes. Their average size is in the nanometer range and they can be easily distributed over the whole body by intravenous administration. Since they are small, they can diffuse into the extravascular media and may be eliminated by glomerular filtration.

The most used paramagnetic cation for this purpose is Gd^3+^. This cation has a high electronic magnetic moment thanks to its seven non-paired 4f electrons. When submitted to a strong static magnetic field, B_0_, its moment is aligned parallel to the direction of the field. Its effective magnetic moment is 7.95 µ_B_. Free Gd^3+^ are nephrotoxic and neurotoxic [27]. For these reasons, they are never used without strong chelation to polydentate hydrophilic ligands (Figure 2) to make them as stable as possible in physiological media.

Other paramagnetic cations, chelated by the same kind of ligands, have also been investigated as potential positive CAs. These cations are mainly from the first series of d-block elements, like Mn^2+^ and Fe^3+^, which have an effective magnetic moment of 5.92 µ_B_, smaller than the 7.92 µ_B_ of Gd^3+^. This magnetization decrease is expected to decrease total relaxivity. Typically, chelating Mn^2+^ by N,N’-dipyridoxylethylenediamine-N,N’-diacetate-5,5’-bis(phosphate) (DPDP) forms a Mn-DPDP complex with a longitudinal relaxivity r_1_ of 1.6 mM^−1^.s^−1^ (T = 25 °C, B_0_ = 0.47 T), smaller than the usual 4 mM^−1^.s^−1^ of monomeric Gd complexes within the same operating conditions [28,29]. Besides Gd^3+^, other paramagnetic lanthanide cations were tested. The most commonly studied is Dy^3+^, which has the largest effective magnetic moment value (10.65 µ_B_) and which has been expected to allow reaching higher longitudinal relaxivity values. In fact, its highest effective magnetic moment and its shortest electronic relaxation time (~10^−13^ s), make it much more investigated for T_2_ contrast enhancement at ultrahigh field [30,31].

The longitudinal relaxivity of all these molecular complexes can be tuned by replacing the conventionally used polydentate ligands with ones having a higher molecular weight increasing thus the total tumbling time. It can be also increased by assembling the paramagnetic centers in polymeric complexes [2]. More sophisticated assemblies can be also built to increase r_1_. They consist of dispersing several monomeric Gd complexes based on diethylene-triamine-pentaacetate (DTPA) or 1,4,7,10-tetraaza-cyclododecane-1,4,7,10-tetraacetate (DOTA) chelators into biocompatible polymers [32,33,34,35,36] or by attaching them around hydrophilic dendimeric structures [37] (Table 1).

**Figure 3:**
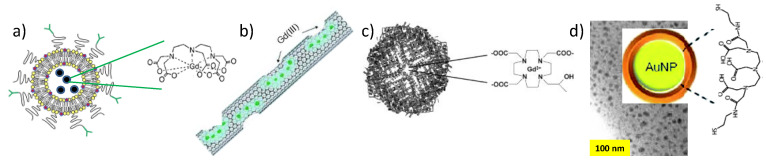
Schematic representation of different monomeric Gd assemblies: (**a**) Liposomes encapsulating Gd-DTPA complexes, (**b**) CNTs, and (**c**) apoferritin compartmentalizing free or coordinated Gd^3+^ ions in their cavities, and (**d**) gold nanoparticles functionalized with Gd-DTPA complexes.

Compartmentalization of free or chelated paramagnetic cations, mainly Gd^3+^, was also explored as chemical strategy to enhance r_1_ (Figure 3). Liposomes [38], carbon nanotubes (CNTs) [39,40,41], or the cavities of certain proteins, like apoferritin [42,43] were used in order to significantly increase the number of Gd-coordinated water molecules and then to enhance the longitudinal relaxivity of the resulting architectures. Unfortunately, despite the very promising physical properties measured on this new generation of probes, their weak stability in biological media compromised their clinical use. Finally, with always the same goal of increasing the number of interacting water molecules, biocompatible inorganic particles, like silica or gold, were decorated by monomeric Gd-chelates (Figure 3), with very encouraging results [44].

The last family of positive CAs is that based on heavy paramagnetic inorganic NPs, which have intrinsically high τ_R_ values. These particles are functionalized by hydrophilic molecules or biomolecules to make them biocompatible. Most of them are 3d- and 4f-block metal oxides like Mn_3_O_4_ [48], MnO [49], and Gd_2_O_3_ [50,51,52]. Their longitudinal relaxivity depends mainly on their size, the spin of the metallic cation and the organic coating, which should allow water to interact with the paramagnetic surface cations. Experimentally, their r_1_ values were found to be of the same order of magnitude as those of molecular CAs (Table 2). Recently, ultra-ultrasmall superparamagnetic iron oxide NPs (UUSPIOs) with a very small size (less than 5 nm) and a very small magnetization (≤10 Am^2^.kg^−1^), have been included in this last class of CAs [53,54]. Their measured r_2_/r_1_ values close to 1 explain their classification. They are not yet commercially available, but they would be an excellent alternative to the more expensive gadolinium-based CAs, currently used and still suspected of toxicity [55].

### 2.2. Negative Contrast Agents

Negative CAs are exclusively superparamagnetic NPs coated with hydrophilic ligands or polymers to improve their ability to form stable aqueous colloids. Their introduction in a solution or a tissue induces local magnetic field gradients, which accelerate the loss of phase coherence of the water proton magnetic nuclear moments, improving MR contrasting at their proximity [26,57].

**Figure 4:**
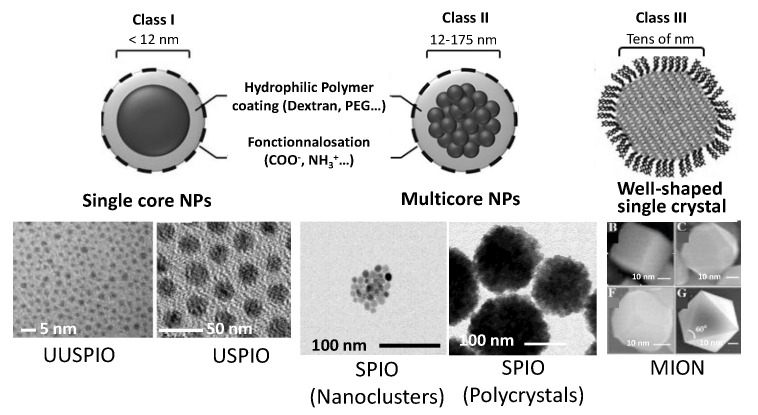
Schematic representation of the three main iron oxide-based contrast agents and selected TEM images.

These CAs are mainly based on iron oxide nanocrystals, which were marketed in the 2000–2010s, under various names (Feridex, Resovist, Sinerem, Lumirem, GastromMARK, Sienna+, Feraheme…) [58]. They are classified according to their average size and their aggregation state (Figure 4). Usually, individually dispersed iron oxide cores with a hydrodynamic diameter below 40 nm are referred as ultrasmall superparamagnetic iron oxides (USPIOs), while multicore clusters or polycrystals with diameters in the 100–200 nm range are known as superparamagnetic iron oxides (SPIO) [59]. There are also well-shaped and highly magnetized iron oxide single crystals, usually referred as monocrystalline iron oxides (MIONs) [60]. This size classification is very important since it affects the total magnetization of the particles and thus the r_2_ value. The larger the particle, the higher the magnetization is and the higher magnetization, the higher r_2_ is (Table 3). It also defines the ability of these particles to cross blood vessels, when they are intravenously administrated. The commercial agent, Sinerem, which belongs to the USPIO class, may for instance cross the damaged brain blood barrier, whereas Endorem, which belongs to the SPIO class, may cross only liver vessels. It is, moreover, strongly uptaken by healthy Kupfer cells but not at all by malignant liver cells, making it particularly useful for liver imaging.

Non-iron oxide NPs have also been considered for MRI contrasting. In most cases, they consist of superparamagnetic Ln substituted manganites, like La_1-x_Sr_x_MnO_3_ [61,62,63] and ferrites, like Zn_1-x_Fe_2+x_O_4_ [64], MnFe_2_O_4_ [65], CoFe_2_O_4_ [66], Zn_1-x_Co_x_Fe_2_O_4_ [67], Zn_1-x_Ni_x_Fe_2_O_4_ [68], and Zn_1-x_Mn_x_Fe_2_O_4_ [69] among others. Exchange-coupled magnetic NPs, commonly called enhanced ferrite nanoparticles (EFNPs), consisting of a metallic core, mainly iron, coated by a ferrite shell, mainly iron oxide, form another class of negative CAs. Their structure allows increasing the total magnetization of the engineered particles and prevents the oxidation of their highly magnetized metallic cores [70]. For comparison, such EFNPs exhibit r_1_ and r_2_ relaxivities of 7.19 and 9.96 mM^−1^·s^−1^ (expressed per particle), respectively, at room temperature for an applied B_0_ of 2.4 T. These values are higher than those for commercial iron oxide-based CAs commonly used in human MR examinations if expressed per particles.

Finally, d-block metal NPs, like Fe or Fe-Co, protected from air and water oxidation, have been tested. These CAs have higher magnetization (typically 185 Am^2^ .kg^–1^ for Fe-based NPs at room temperature) than all SPIO- and ferrite-based particles (typically 60–90 Am^2^.kg^−1^ at room temperature) [71]. As a consequence, despite their very reduced size, their r_2_ and r_2_ /r_1_ values are expected to be very high (Table 4).

An important issue of negative CA design is their surface modification to make them biocompatible for in vitro and in vivo uses. This modification usually involves: (i) the synthesis of hydrophilic polymer brushes from the CA particle surface (‘grafting-from’ reactions), using different coupling ligands (silane, carboxylate, phosphonate…) [75,76]; (ii) the attachment of preformed polymer brushes at the surface of the particles by different surface reactions (click chemistry, diazonium chemistry…) [77]; (iii) the self-assembly of polymer chains with the magnetic particles, based mainly on electrostatic interactions [78], leading to core@shell [79] or embedded [80,81] morphologies; (iv) the grafting of specific molecules [82,83] or biomolecules [6,84] with targeting ability. This list is, of course, not exhaustive but it gives an idea of the further research directions in the field of negative CAs. The polymers most used are polysaccharides (Dextran, Alginate, Chitosan…), polyethyleneglycols (PEG), polyvinylpyrrolidone (PVP), and polyvinyl alcohol (PVA), polycaprolactone (PCL), polyacrylic acid (PAA), certain polypeptides, and fatty acids, due to their ability to increase the aqueous colloidal stability of the particles [72]. The targeting biomolecules can be specific proteins like transferrin [84] and TRAIL [6], short peptides like RGD [85,86] and Tat [87,88], or just small molecules like folic acid [83] and dopamine [82].

Surface modification may also involve an inorganic coating or embedding with inert and hydrophilic silica [89,90,91] or hydroxyapatite [92], which may also serve as matrix for drugs, or radioisotopes, transforming the initial CAs into theranostic agents.

## 3. Optical Imaging Probes

OI applied to living organs or cells has become a fundamental tool for imaging functional lesions in vivo and in vitro. It offers the possibility of real-time spatio-temporal monitoring of biological processes in a non-invasive way. This technique takes advantage of the phenomenon of intrinsic luminescence induced by endogenous biological matter or by exogenous species introduced near the biological structure to be imaged. Interestingly, certain exogenous dyes, in addition, to be able to illuminate a given area of their biological environment, they are able to express its physicochemical state, acting as metabolism marker. Their optical signal may be thus correlated to the variation of biological parameters like the pH and the calcium concentration [93,94,95,96].

**Figure 5:**
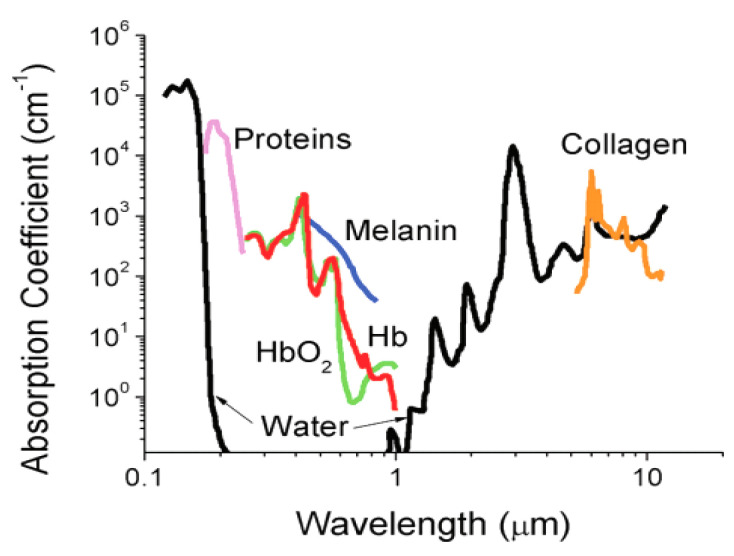
Absorbance of various tissue and blood components from 200 nm to 10 μm. The optical imaging window ranging from 650 to 1450 nm represents the range where tissue penetration is greatest, HbO_2_ and Hb referring to oxygenated and deoxygenated hemoglobin. Reproduced from [97] with permission from ACS, 2012.

Nowadays, OI is routinely used for in vitro observations, thanks mainly to fluorescence microscopy. However, its use for in vivo diagnostics is still in progress and to date, only endoscopic light exciting or detecting systems are used. Indeed, the incident as well the emitted photon may be: (i) reflected by the surface of the tissue; (ii) absorbed by the tissue, losing its energy; (iii) finally, scattered by the tissues. As a consequence, a loss of coherence of the light occurs and it becomes impossible to know where the photons are going out or coming from limiting the OI operating spectral range to the body transparency window namely between 600 and 1000 nm (Figure 5) [97]. Anyway, technological advances in the field of electronics, with the development of very high-resolution, ultra-sensitive and very small CCD cameras as well as optical fibers allowed the fabrication and the commercialization of endoscopic imaging systems, coming very close to the target tissue and detecting directly, inside the body, the emitted photons. Systems adapted to a conventional bronchoscope, in which the conventional beam is replaced by blue laser light (442 nm), have been thus successfully used to image the lungs and bronchi. The emitted light has been collected by means of an intensified CCD camera, connected to the image beam of an endoscope. Optical imaging devices using endoscopic pathways and allowing the measurement of the autofluorescence of some endogenous porphyrins have been also proposed for urology, gynecology, and otorhinolaryngology [98,99,100]. More recently, two low energy photon excitation technology has been developed, in replacement of the conventional single high energy photon excitation one, making the dye photoexcitation less-harmful and deep-penetrating, even carried out outside the body [101].

Exogenous dyes usually produce light locally after a photonic excitation. Each dye can be defined by its excitation and emission spectra, its lifetime (the time taken by the fluorophore to emit after the cessation of the excitation), and its quantum yield. In practice, the excitation wavelength chosen to trigger the reaction of a specific dye may lead to a ‘parasite’ luminescence of endogenous dyes, which must be discriminated to avoid OI artifacts. For such a purpose, light information is often processed as a function of time. Indeed, as soon as the excitation ceases, the fluorescence gradually fades, according to a variable time constant which depends on the nature of the fluorophore. If the exogenous dyes exhibit long relaxation times, longer than those of endogenous ones, one has to wait for the extinction of the endogenous fluorescence before opening the camera shutter [102,103]. Reversely, if their relaxation times are shorter, the camera shutter must be closed quickly, and the endogenous fluorescence contribution must be subtracted from the recorded signal.

Finally, the quantum yield Φ of an exogenous dye, measures the ratio of the number of photons emitted to the number of photons absorbed during the lifetime of its excited state. It is usually defined by the equation

Φ = k_r_/(k_r_ + k_nr_) (2)

where k_r_ and k_nr_ are the rate constants of its radiative and non-radiative de-excitation after absorption of the incident photon. In practice, it is determined by comparing the absorption and emission spectra of a reference compound, excitable at the same wavelength as the dye and whose emission covers the same range as that of the compound of interest. The higher the Φ, the more efficient the exogenous dye is. In contact with physiological media, Φ may decrease drastically, reaching luminescence extinction. In the case of molecular dyes, this extinction is mainly due to the chemical reactivity of the fluorophores with free oxygen-based radicals. In the case of solid ones, it is much more due to the interaction of their organic coatings with the available bio-organics, leading to photon reabsorption. These differences justify the classification of all the optical imaging probes into two classes: molecular dyes, including organics and lanthanide chelates, and inorganic, mainly solids including PPs, QDs, CDs, DCNPs and UCNPs. However, for our purposes, we will classify all these agents into two main families, Ln-containing and Ln-free dyes.

### 3.1. Ln-Free Dyes

**Figure 6:**
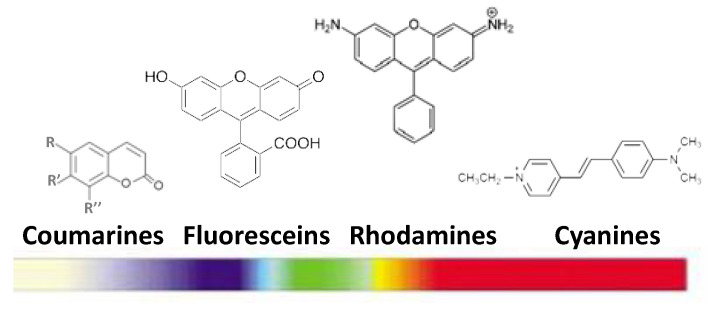
Main families of organic chromophores and their emission wavelength ranges.

This luminescent agent family is rich. Their major representatives are organic chromophores. Briefly, they consist of natural or synthetic conjugated organic molecules. Their excitation is very fast (10^–17^ s) and their excited lifetime is of few nanoseconds [8]. Their radiative relaxation leads to the emission of a photon of energy lower than that of the one absorbed (Stoke displacement). Most of these molecular dyes are commercialized: coumarins, fluoresceins, rhodamines, and cyanines. Their absorption and emission cover the entire near-UV to near-IR spectral range (Figure 6). Their quantum yields are relatively low, about 10–15% in visible light and 2–4% in IR [104,105].

**Figure 7:**
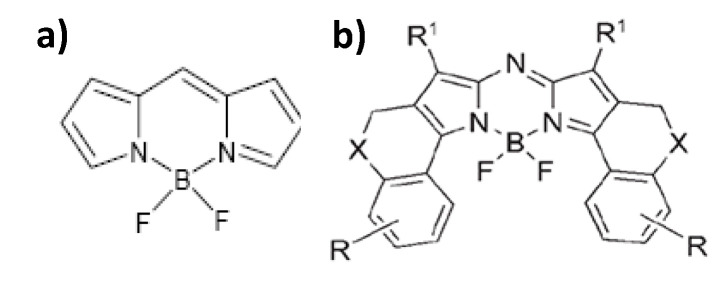
(**a**) Bodipy dye and (**b**) its aza derivative. Reproduced from [106], with permission from John Wiley and Sons, 2006.

These last years, dipyromethene borane derivatives, commonly known as bodipy, have emerged. Thanks to their higher quantum yields, approaching 100% for their aza-based compounds (Figure 7), and their emission wavelengths range between 520 and 670 nm [106] with a life-time of some nanoseconds (from 3.9 ns in water to 5.7 ns in methanol), they have attracted a lot of interest. They suffer nevertheless from certain drawbacks: (1) their Stoke displacement is low, making it difficult to filter the scattered incident light (at the excitation wavelength of the system) and the emission signal; and (2) their absorption and emission bands are broad, making it awkward to use different bodipy markers simultaneously [106].

**Figure 8:**
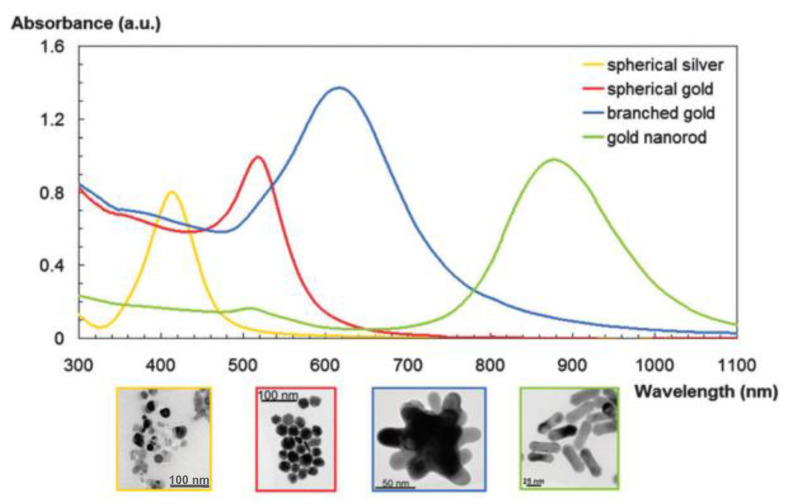
SPR feature of gold and/or silver NPs of different shapes. Reproduced from [111], with permission from RSC Publisher, 2012.

Ln-free dyes class includes nanosolids, PPs and QDs. PPs, mainly gold NPs, exhibit tunable optical properties including Mie scattering, surface plasmonic resonance (SPR), surface-enhanced luminescence and surface Raman scattering. They are used for high-sensitivity and high-resolution optical imaging [107,108,109]. Their surface plasmon resonance which corresponds to the interaction with the light of the free conductive electrons on their surface, is the major feature. It causes enhanced absorption and scattering intensities at the SPR wavelength. The intensity and position of the SPR can be controlled by the size and the shape of the PPs but also by the dielectric constant of their surrounding medium. The SPR wavelength is thus red-shifted with increasing the size of PPs, changing their shape from an isotropic to an anisotropic morphology (nanorods [110], nanostars [110,111]… Figure 8), or coating them with dielectric materials of high refractive index (iron oxide [112,113], polyvinyl pyrrolidone (PVP) [114,115]…).

Besides, PPs show superior photostability, which means that they can serve as multi-colored optical probes and sensors for in vivo real-time imaging. Additionally, the non-toxic and chemically stable ones, like gold NPs, can be delivered into living organisms non-invasively. They can be easily functionalized by various biotargets thanks to metal–SH bio-conjugation, making them, gold PPs in particular, of primary importance for in vivo and in vitro OI.

**Figure 9:**
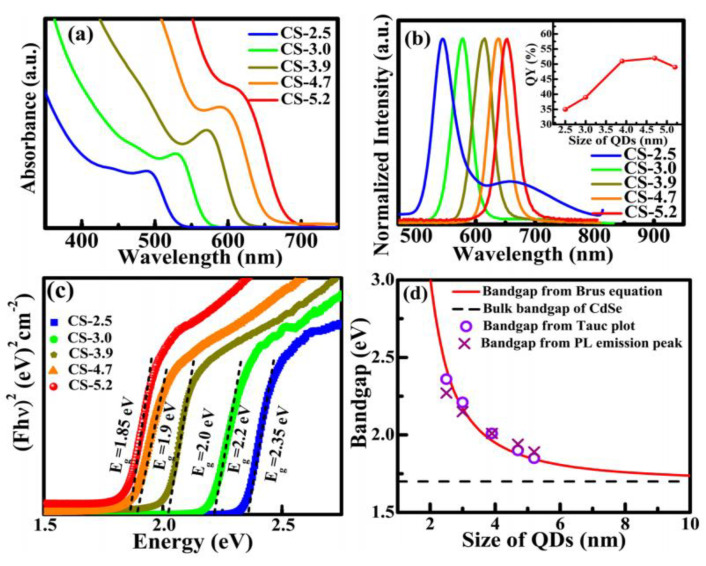
(**a**) Absorption spectra, (**b**) normalized emission spectra, (**c**) Tauc plot obtained from diffuse reflectance spectra, and (**d**) the comparison of the experimentally determined bandgap of CdSe QDs with theoretically calculated values using Brus equation. Te emission spectra were recorded using an excitation wavelength of 400 nm. The inset in (**b**) shows the value of the corresponding quantum yield of different sized QD and the fat line (at 1.7 eV) in (**d**) indicates the bulk band gap value of CdSe. The band gap is calculated experimentally from the Tauc plot and from the PL emission peak position (**d**). Reproduced from [117], with permission from Nature Publishing Group, 2018.

The last Ln-free dye type is that of QDs. QDs exhibit a strong emission resulting usually from the excitation by photons of energy hυ_ex_ higher than their band gap *E_g_*. This emission proceeds as radiative recombination of the photogenerated hole (in the valence band) and the electron (in the conduction band) pair (exciton). The radiative recombination of excitons can be direct (band-to-band) or indirect. In the latter case, lattice defects or impurities (doping), associated with levels of energy located in the gap, act as recombination centers, and an intermediate step is involved in the return of the excited electron from the conduction band to the valence band of the semiconductor. The surface defects of the nanocrystalline semiconductor can be a preferred site for recombination. One can very well imagine that this surface-related mechanism is important, maybe the most important, to the point of greatly modulating the luminescence properties of this kind of dye. Additionally, due to quantum confinement effect, ultrafine QD bands may split into discrete levels and their band gap increases when the radius of the nanocrystals *r* decreases, leading to *E_g_* values higher than those of bulk counterparts [116]. The wavelength of the band-to-band radiative de-excitation decreases, making any QD-based biolabeling size-dependent (Table 5, Figure 9).

In practice, QDs are passivated by hydrophilic organic ligands, to improve the dispersion of the particles in aqueous media and minimize the non-radiative recombination of the photogenerated exciton by interaction with the external environment. It should be noted that organic ‘passivation’ is not always enough to avoid fluorescence extinction. As an alternative, each QD can be replaced by a semiconductor-semiconductor core–shell counterpart, whose quantum efficiency may exceed 50% [126,129,130,131]. Of course, the engineered semiconducting hetero-junctions have to be finally surrounded by a hydrophilic organic [132,133,134] or inorganic (mainly silica) [135,136] thin layer to make them soluble in physiological media. 

Despite all these very interesting optical properties, QDs have been seldom employed in OI. Their toxic heavy metals composition with the risk of release of these elements makes their use still controversial. Studies are underway to determine the dose effects for each QD family, based on their size, chemical nature, and surface state [137,138,139,140]. Only efficient clearance [141] may promote their application in clinic.

Recently a new class of metal free semiconductive NPs has emerged. It is that of surface-passivated carbon dots (CDs). They have successfully been used as in vitro [16] and in vivo [142] biolabels. To date, CDs are considered as safe materials, non-toxic towards different cell lines even at high concentrations [143].

### 3.2. Ln-Based Dyes

**Figure 10:**
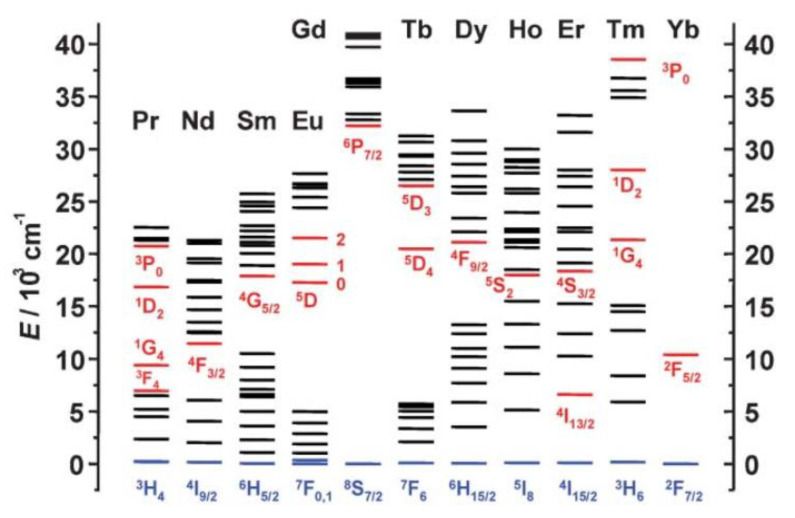
Partial energy diagrams for the lanthanide aquo ions. The main luminescent levels are drawn in red, while the fundamental level is indicated in blue. Reproduced from [147], with permission from RSC Publisher, 2005.

The optical properties of Ln cations derive from the electronic transitions of the excited state, populated under illumination, towards the ground state (f–f transitions), either directly or indirectly by transiting towards excited levels of lower energies (Figure 10). As a consequence, their emission bands are narrow and of specific energies [144]. Moreover, their excitation life-times are longer, from a few microseconds to several milliseconds, than those of organic chromophores. This property makes it easy to discriminate between their luminescence signal and that of endogenous dyes [145,146].

As biolabels, they are used as hydrophilic complexes (molecular dyes) or as luminescent ceramic NPs.

### 3.3. Molecular Dyes

Ln cations are chelated to hydrophilic polydentate ligands to form stable complexes, for which the energy of the involved f-f- transitions are weakly affected by either the cation environment or the experimental measurement conditions [144,148]. Timely, to achieve their clinical use, these molecular edifices are upgraded by the covalent grafting of labeling functions [149].

**Figure 11:**
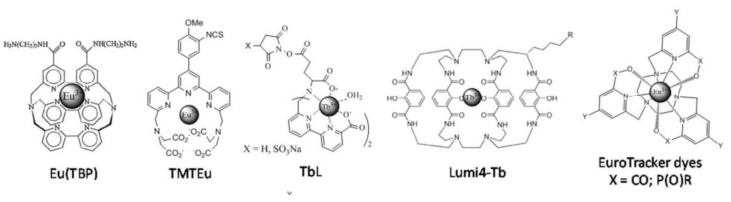
Schematic representation of a luminescent lanthanide label. Reproduced from [149], with permission from RSC Publisher, 2016.

The design of this family of molecular dyes involves also two main improvements, in relation to some intrinsic limitations. Indeed, the photoluminescence intensity of Ln cations is weak [147]. The electric dipolar f-f transitions, at the origin of the luminescence spectrum, are forbidden by the Laporte and sometimes spin multiplicity rules [149]. It results that Ln^3+^ have extremely low extinction coefficients (few units M^−1^.cm^−1^ in the best cases) and their direct excitation through these transitions is difficult and consequently their photoluminescence is weak. This can be overcome by sensitizing the lanthanide cations through a covalent modification of their ligand with some aromatic molecules, called antenna, (Figure 11) having an appropriate electronic structure for fluorescence resonance energy transfer (FRET) [150]. Secondly, the risk of their photoluminescence quenching by the OH, NH, and CH oscillators in their inner coordination sphere is not negligible at all [149,151]. To minimize this effect while obtaining extremely stable water-soluble edifices, Ln coordination must be balanced between the strength of the electrostatic interactions between Ln^3+^ and the ligands and steric or electrostatic repulsion interactions between the ligands around the cations, while filling as much as possible the coordination sphere, avoiding water bonding. There are essentially three strategies for such a purpose. The first is to use highly pre-organized ligands featuring macrocycles such as triazacyclononane or 1,4,7,10-tetraazacyclododecane (Cyclen) [152,153,154]. The second strategy is to provide numerous negatively charged functions such as carboxylates or phosphonates [154,155] (Figure 11).

### 3.4. Solid Inorganic Dyes

**Figure 12:**
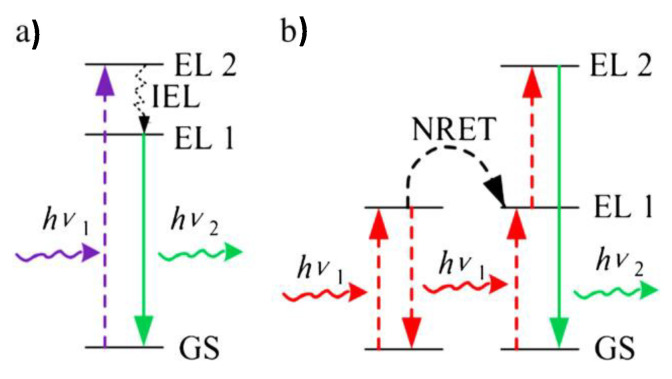
Illustration of (**a**) down-conversion and (**b**) energy transfer up-conversion mechanism. IEL: internal energy loss; GS: ground state; EL: energy level; NRET: non-radiative energy transfer; hν_1_: incident light; hν_2_: emission light. Reproduced from [17], with permission from ELSEVIER Publisher, 2012.

Ln-doped up-conversion and down-conversion luminescent ceramic nanocrystals (UCNPs and DCNPs) have been seriously considered for OI application. With an appropriate surface modification, they may form an interesting class of inorganic dyes, particularly UCNPs. In practice, they consist of a transparent crystalline host lattice that accommodates the dopants. For high-yield light emission, the host lattice must closely match that of the dopant ions, have low phonon vibration energies, and good chemical stability [156,157]. Based on these criteria, the most commonly used host lattices for the synthesis of UCNPs are fluorides [158,159,160], oxides [161,162,163], and sometimes phosphates [164,165,166] (Table 6). Fluoride matrixes are the most efficient UCNPs [167,168,169]. These are commonly doped with two luminescent cations: the activator, emitting visible light and the sensitizer, absorbing the photoexcitation, and transferring the required energy to the activator (Table 6). To minimize cross-relaxation energy loss, the concentration of the sensitizer is relatively high (~20 mol. %), while that of the activator is low (below 2 mol. %) [169,170,171,172]. In this mechanism, higher-energy photons are emitted by sequential absorption of lower-energy photons (Figure 12) [17,173], leading to very strong emission and increasing the optical detection sensitivity.

UCPNs exhibit great advantages over other types of fluorescent materials. Thanks to their NIR excitation, they allow enhanced tissue penetration depths. Excitation by the light of such low energy avoids DNA or RNA photo-damage. Moreover, the chemical composition of the host and the nature of the dopant improve their stability against photobleaching, photoblinking, and photochemical degradation, and significantly reduce their cytotoxicity [17]. Finally, their optical properties do not depend on their size and shape. The only requirement for their in vivo use is that they must be small enough and almost uniform in size. As the particles are small, they are able to diffuse inside the body and to reach the target organs.

Finally, the judicious surface functionalization of UCNPs allows in vitro and in vivo, targeting and molecular events detection, making these objects not only useful for the selective detection of cells, but also for the elucidation of biological processes. For instance, polyethyleneimine-coated NaYF_4_:Yb/Er particles conjugated with folic acid were employed for in vitro imaging of HT29 adenocarcinoma cells and OVCAR3 ovarian carcinoma cells [180]. NaGdF_4_:Er/Yb particles functionalized with heparin and basic fibroblast growth factor (bFGF) molecules were also employed for in vitro imaging of HeLa cells. Heparin molecules not only provided water dispersibility, but their interaction with the growth factor resulted in the required conformation of bFGF to interact with receptors on the cell membrane of epithelial cancer cells, optimizing the targeting abilities of the luminescent nanoprobes [181].

## 4. Ln-Based Nanoprobes for Dual Magnetic and Optical Imaging

**Figure 13:**
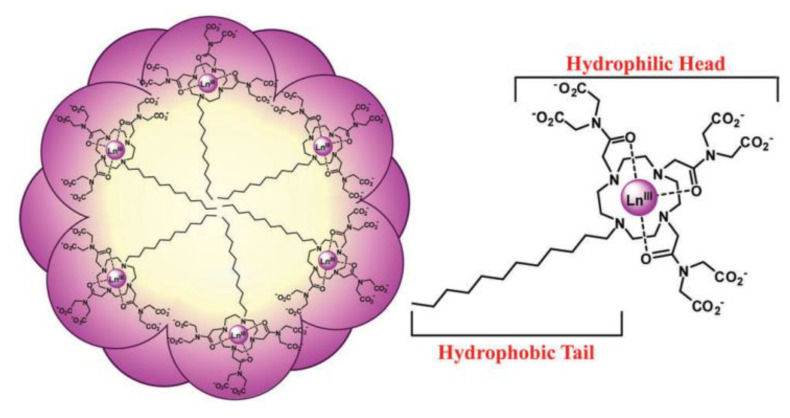
Schematic representation of the spherical micelles formed in aqueous solution with intertwined hydrophobic tails in the interior and hydrophilic head groups at the exterior. Ln(III) = Eu(III), Gd(III), or Lu(III). Reproduced from [182], with permission from RSC Publisher, 2016.

There are several ways to introduce bimodality in an imaging probe. They depend on whether the magnetic and the luminescent species are distinct, just assembled in a common compartment or if they form a single object. On this basis, there is a first classification of the bimodal probes: probes in which the magnetic and luminescence properties come from two distinct species, brought together only for this purpose, and probes in which the magnetic and luminescence properties come from the same chemical object. In vivo, the former dual probes may be dissociated and impacted by microenvironmental conditions (pH, enzymatic activities), the optical label and the magnetic dipole, becoming tracked separately, making the later cases much more suitable for the desired application. Among these architectures, there are those constructed using micellar chemistry. In practice, luminescent and paramagnetic Ln chelates are assembled within the same micellar structure [182,183] as illustrated in Figure 13. There is also the possibility to interact simultaneously with molecular chromophores and paramagnetic chelates with selected nanocontainers like zeolites [184] or CNTs [185].

Let us focus on the ‘two-in-one’ bifunctional probes. There are several types: all-molecular, hybrids, and all-inorganics. The first sub-family consists of polynuclear complexes, involving both magnetic and luminescent lanthanide cations. They can be used only as positive MRI contrast agents, and their optical properties are mainly of the down-conversion type. The second sub-family is a kind of core–shell structure made from a luminescent core, typically lanthanide-doped UCNPs, and a shell containing paramagnetic Gd^3+^ complexes and reversely a magnetic one, mainly iron oxide NPs, and a shell of luminescent Ln^3+^ complexes covalently bonded to the former. Depending on their architecture, they can proceed with up- or down-conversion luminescence while leading to positive or negative MRI contrasting. They are again small and offer more versatility for both in vivo and in vitro imaging. Within these sub-classes, there are also dual-Lanthanide-chelated silica particles [186,187,188,189]. The chelates can be embedded into the silica core or just attached to its outer surface. The former configuration is much more appropriate for dual imaging since it allows direct contact between water molecules and paramagnetic cations as it limits light scattering with the silica matrix. Finally, the third sub-family includes two types: (i) doped single crystals like Er-Tr or Ho doped superparamagnetic iron oxide Fe_3_O_4_ NPs [190,191], Eu doped paramagnetic gadolinium oxide Gd_2_O_3_ NPs [192,193], or Gd co-doped UCNPs and (ii) crystalline hetero-nanostructures like Fe_3_O_4_@LaF_3_:Ce,Tb [194,195], NaYF_4_:Yb^3+^-Er^3+^@Fe_3_O_4_ [196] or NaYF_4_:Yb^3+^-Er^3+^@NaGdF_4_ [197,198] core–shell NPs. Even if both kinds of probes combine high sensitivity of time-resolved fluorescence and high spatial resolution of MRI, there are some reports pointing out the possibility of a host lattice induced optical quenching in the former [199], making the latter much more studied. There is a vast range of possibilities for this sub-family of probes thanks to the thousands of combinations that can be made between the core and the shell, while remaining the resulting architectures always small in size for improved in-body diffusion.

### 4.1. Molecular Dual Probes

**Figure 14:**
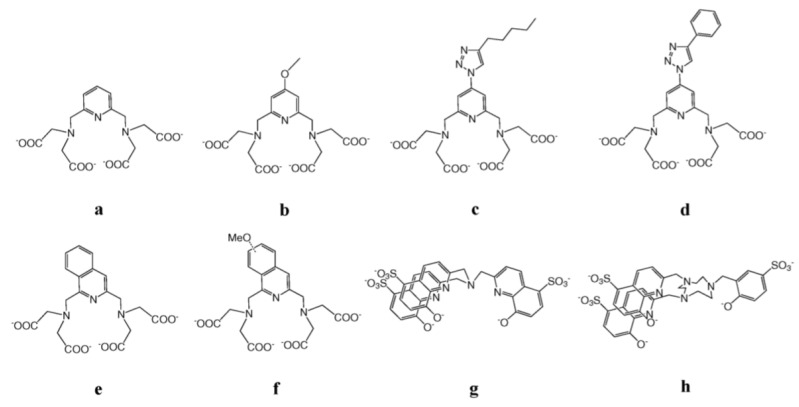
Structure of pyridine-based ligands used to chelate both paramagnetic and luminescent lanthanides for molecular bimodal imaging probes.

Molecular dual probes consist of polynuclear complexes, in which both luminescent and paramagnetic lanthanides are simultaneously chelated. The design of such architectures is a real challenge for coordination chemists. Whereas the presence of at least one inner-sphere water molecule is required for good MRI efficiency, it is not for non-quenched luminescence imaging [200]. Some synthetic strategies were nevertheless proposed to overcome this drawback. A versatile pyridine-based scaffold for Ln^3+^ complexation was for instance constructed (Figure 14a,b) [201]. In this structure, the paramagnetic Ln cations, particularly Gd^3+^, are bishydrated, giving good MRI efficacy (a relaxivity r_1_ of 6.21 mM^−1^·s^−1^ at 500 MHz and 25 °C per Gd^3+^) while the luminescent ones, particularly Nd^3+^, are sensitized by the aromatic pyridine moieties, tacking advantage from the resulting ‘antenna effect’ to improve their photoconversion rate, compensating the quenching caused by the two inner-sphere waters.

Other derivatives of these pyridine-based ligands were prepared for the same purpose. They were obtained by extending the pyridine group with a triazole ring (Figure 14c,d) [151] or by replacing it by an isoquinoline (Figure 14e,f) [202]. The former compounds gave quantum yields of 0.01% and 0.02% for the NIR-emitting Nd(III) and Yb(III) complexes, respectively, while the latter gave slightly higher yields of 0.013–0.016% and 0.028–0.040% for the same metal cations. In all cases the relaxivity of Gd(III) complexes was of the same magnitude, ranging from 6 to 8 mM^−1^.s^−1^ at 20 MHz and 37 °C. NIR emission could also be observed after complexation of the tripodal hydroxyquinolinate ligand (Figure 14g) to Nd(III) or Yb(III) or that based on triazacyclononane and 8-hydroxyquinolinate/phenolate binding units (Figure 14h), which are considered to be good sensitizers for luminescent lanthanide cations, leading to quantum yields around 0.02%, as for the pyridine-based complexes. Interestingly, these two ligands are not equivalent in their MRI applications. Indeed, while the latter has a large r_1_ value for its bishydrate Gd(IIII) complexes of about 9.1 mM^−1^.s^−1^ at 20 MHz, as a result of a long rotational correlation time, fast water exchange and slow electronic relaxation, the former has not, due to a slow water exchange [203].

**Figure 15:**
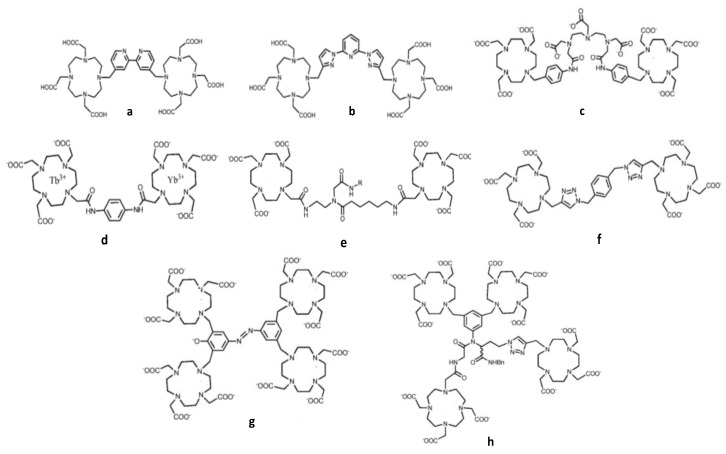
Structures of DOTA-based ligands used to chelate both paramagnetic and luminescent lanthanides for bimodal molecular imaging probes.

Encouraging results were also obtained by replacing linear polydentate ligands with cyclic ones, involving aromatic moieties as bridging ligands (Figure 15) [204,205,206,207,208,209]. The resulting macromolecule exhibits a characteristic slow tumbling with high longitudinal relaxivity, due to the formation of nanosized aggregates. Their luminescence quantum yields remain relevant taking advantage of the antenna effects of their aromatic moieties.

To date, the given above polydentate ligands have been principally bonded to only one type of lanthanide cations, only luminescents or only paramagnetics. The combination of the two types of cations would provide cocktail of paramagnetic/luminescent Ln^3+^ complexes, assumed to be good candidates for the development of efficient bimodal agents. Very promising results have been obtained by using these ligands for f–d heteropolymetallic complexes, like Gd^3+^ and Ru^2+^ or Gd^3+^ and Ti^4+^ ones [210,211].

**Figure 16:**
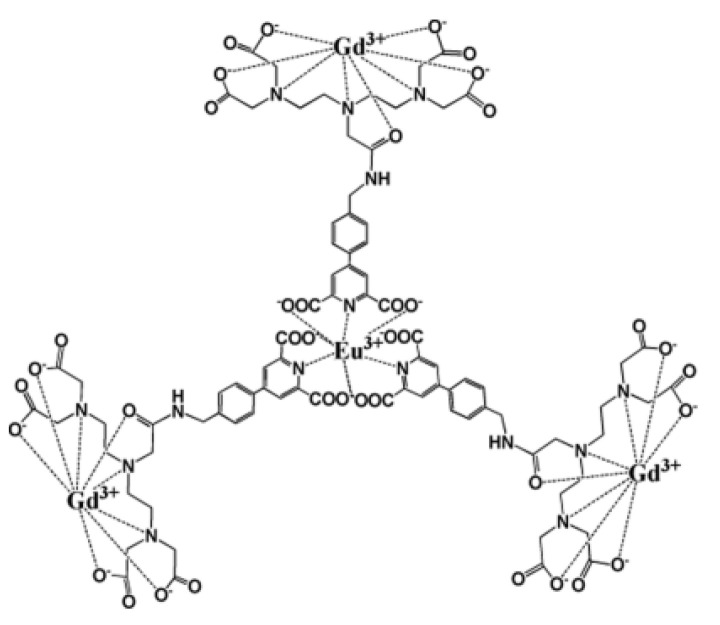
Schematic representation of the (GdL)_3_Eu, containing. Reproduced from [212], with permission from ACS Publisher, 2014.

Respecting this general chemical approach, more specific architectures started to be proposed these last 5–6 years, based on the spatial separation of the luminescent and the paramagnetic cation compartments. The f-f molecular architecture built by Debroye et al. and abbreviated as (GdL)_3_Eu, is a good example (Figure 16). In this structure, three DTPA have been chosen as Gd^3+^ chelating units, and have been linked to a central Eu^3+^ chelate, consisting of a para-substituted pyridine-2,6-dicarboxylate derivative, via an amide bond, achieving bright-red luminescence with a quantum yield of 10% and an enhanced longitudinal relaxation rate of 31 mM^−1^.s^−1^ per molecule at 40 MHz in water at 310 K [212].

**Figure 17:**
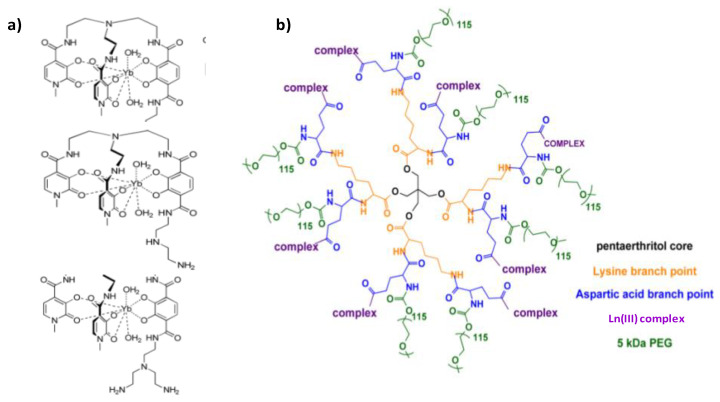
Structures of (**a**) Ln(III) complexes for MRI and NIR imaging agents and that of (**b**) the esteramide dendrimer scaffold. Reproduced from [213], with permission from ACS Publisher, 2012.

This spatial separation strategy was also successfully employed to build a new class of dual probes that of lanthanide complex dendrimer conjugates. The paramagnetic and luminescence properties of Dy(III) and Yb(III) were thus exploited by chelating them with the all-oxygen-donor-hexadentate ligand TREN-bis(1-Methyl)-3,2-HOPO-TAM-NX (X = 1, 2, or 3), in which TREN, HOPO, and TAM correspond to tris(2-aminoethyl)amine, hydroxypyridinonate, and tris(hydroxyethyl)aminomethane, respectively. The resulting complexes were subsequently conjugated to the esteramide dendrimer (Figure 17) to improve bioavailability, solubility, and relaxivity [213]. In these structures, all the lanthanide ions are peripheral, accessible to free water. Since they are also bishydrated their MRI efficacy is good. The large scaffolding and mass of the dendrimer, to which up to eight complexes may be covalently conjugated, increases the tumbling time of the complex and contributes also to the improvement of the MRI contrast. The r_1_ value was 7.60 mM^−1^.s^−1^ at 27 °C for an applied static field of 1.41 T. The presence of water in the inner sphere of the Yb^3+^ complexes, of course, reduces their NIR luminescence quantum yield, but does not extinguish it. In mouse serum, the value was 0.17% [213]. Despite their ~40 kDa molecular weight, these probes have been excreted by glomerular filtration over several days making the engineered probes quite safe [213].

**Figure 18:**
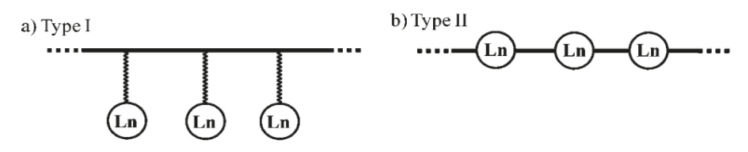
Two different strategies for the introduction of lanthanide binding sites into molecular multifunctional probes. The chelating units are (**a**) connected to the periphery (type I) or (**b**) in series through bridging ligands (type II). Reproduced from [215] and [216], with permission from ACS, 2008, and RCS, 2011, respectively.

**Figure 19:**
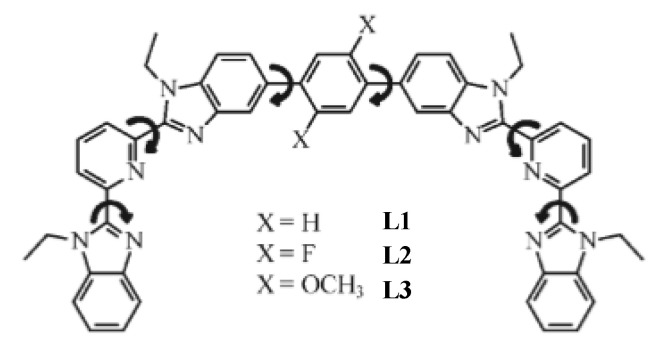
Structure of ligands L resulting from Suzuki–Miyaura coupling of two tridentate 2,6-bis(benzimidazole-2-yl)pyridine binding units at the 1 and 4 positions of a rigid phenyl spacer. Reproduced from [214], with permission from ACS, 2011.

The last strategy for engineering all-molecular bimodal probes is based on organic light-emitting diode (OLED) and lighting device technology. It uses oligomers in which specific lanthanide binding sites can be attached to a peripheral structure (Figure 18a) or connected in series through bridging ligands (Figure 18b) along the oligomer chains. The general idea of this strategy consists in exploiting well identified organic polymer scaffolds for the selective sequestration of luminescent and paramagnetic lanthanides, separating them from each other, and keeping the water molecules bonded to the paramagnetic ones away from the complexation sphere of the luminescent ones [214]. Oligomeric ligands, L, were obtained by coupling two tridentate 2,6-bis(benzimidazole-2-yl)pyridine binding units at the 1 and 4 positions of a rigid phenyl spacer (Figure 19). Because of the small number of torsional degrees of freedom imposed by the polyaromatic scaffold, L was reacted with Ln(hfac)_3_ salts, where hfac- is the bidentate hexafluoroacetylacetonate anion, leading to a linear saturated single-stranded complex [Ln_2_(L)(hfac)_6_]. In these architectures, there are no water molecules directly attached to the lanthanide centers. The hydrophilic character of the ligands involved, particularly hfac, allowed their indirect attachment through hydrogen bonding, conserving a certain magnetic effect of the chelated paramagnetic cations on the surrounding water molecules.

### 4.2. Hybrid Probes

Hybrid probes are produced by combining a luminescent inorganic particle with molecular paramagnetic chelates or a paramagnetic or superparamagnetic inorganic particle with molecular luminescent chelates, in a core–shell type structure.

**Figure 20:**
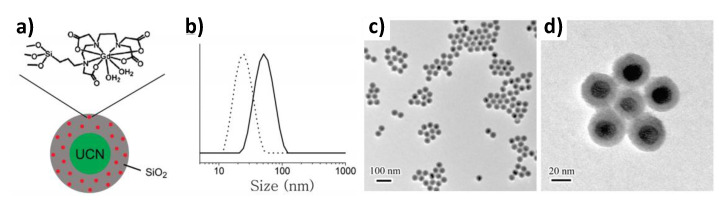
(**a**) Schematic illustration of single NaYF_4_@Si-DTTA-Gd^3+^ NPs. (**b**) DLS data of NaYF_4_ NPs before (dashed line) and after Si-DTTA-Gd^3+^ coating (solid line). (**c**,**d**) TEM images of NaYF_4_@Si-DTTA-Gd^3+^ NPs at different magnification. Reproduced from [19], with permission from ACS Publisher, 2009.

In the first case, Ln-doped UCNP cores have been conjugated to Gd complexes, as reported by Carron et al. Citrate-capped 20 nm Yb^3+^- and Tm^3+^-doped NaGdF_4_ particles were reacted with a mono-amino derivative of Gd-DOTA, leading to multiple paramagnetic surface centers, with improved tumbling time and longitudinal relaxivity. The authors reported a r_1_ value of 25 mM^−1^.s^−1^ per Gd^3+^ ion at 60 MHz and 310 K, seven times larger than that of the Gd-DOTA precursor, which is 3.23 mM^−1^.s^−1^ under the same conditions [217]. Moreover, the excitation of the nanoconstructs in the water at 980 nm resulted in an intense up-converted emission of Tm(III) at 800 nm. To increase the chemical stability and the biocompatibility of this type of dual probes silica coating was used. Typically, UCNPs were first prepared and then coated by a silica thin layer embedding Gd-DTTA complexes as illustrated in Figure 20 [19].

Conversely, about 10 nm-sized USPIOs particles were decorated with Eu(III) ions encapsulated in a DO3A organic scaffold. The organic ligand was modified to bear an ethoxysilane group which allows its attachment by sol-gel chemistry to the surface of an iron oxide crystal [218]. The red luminescence emission of Eu(III) was found at 614 nm. Relaxometric studies showed a r_2_ and a r_2_/r_1_ value of 114.8 mM^−1^.s^−1^ and 18.9 (per iron atom), respectively, at 60 MHz and 27 °C in water, making them particularly valuable for negative MRI contrasting.

**Figure 21:**
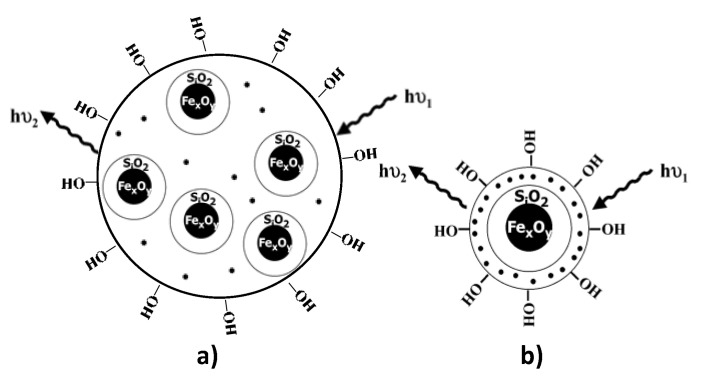
Iron oxide NPs are embedded in a mesoporous silica matrix, in which lanthanide cations are dispersed, within (**a**) a multicore and (**b**) a single core morphology.

To improve the biocompatibility of this type of bimodal probes, mesoporous silica embedding of the iron oxide NPs was achieved, the silica matrix containing the luminescent lanthanide complexes. Two morphologies were largely explored: magnetic multicore [219] and single core [220] morphologies (Figure 21), the latter being much more suitable than the former for the desired application due to the strong silica photo-scattering. By confining the lanthanide-based molecular dyes at the extreme surface of the silica shell, photo-scattering may be avoided or at least minimized improving thus the OI capability of the resulting dual probes. Moreover, the size of the latter is significantly reduced compared to that of the former, meaning better in-body diffusion after intravenous administration. Of course, within these embedded multifunctional nanostructures, superparamagnetic iron oxide NPs can be replaced by perovskite magnetite or spinel ferrite oxide NPs.

### 4.3. All-Inorganic Dual Probes

The last category of single objects for dual MRI and OI applications consists of all-inorganic particles. These are of two types, single crystals, combining both optical and magnetic properties, or core–shell crystalline structures concentrating each property in one compartment, the core or the shell.

#### 4.3.1. Single Nanocrystals

It is possible to have light sources and magnetic dipoles in a same matrix material by inserting the desired components into a same nanocrystal. These probes are expected to offer high photostability, a narrow emission band, and a broad absorption band, combining the high sensitivity of time-resolved fluorescence with the high spatial resolution of MRI. However, to date, most of the bifunctional nanocrystals synthesized to consist of down-conversion emitters and iron oxide NPs. In addition to the fluorescence quenching by iron oxide NPs, down-conversion fluorescence has some intrinsic limitations, such as autofluorescence, low light penetration into the biological tissue, and photon damage risks to the biological specimen. Tb-doped γ-Fe_2_O_3_ nanocrystals, combining superparamagnetism and luminescence, were prepared and evaluated [221]. Functionalized by amino groups they were dispersed in water as a stable colloid. They exhibited green photoluminescence but only under UV excitation (235 nm). This feature prevents the use of such probes for magnetic–fluorescent bimodal in vivo imaging and restricts them to in vitro imaging. The same conclusions can be made concerning Dextran-coated Eu-doped (5 mol%) ultrasmall iron oxide nanocrystals (hydrodynamic diameter between 20 and 40 nm) [191]. Indeed, despite interesting superparamagnetic behavior with relatively high r_1_ and r_2_ relaxivity values (15.4 and 33.9 mM^−1^.s^−1^ in the water at 0.47 T and 37 °C, respectively), their red photoluminescence can not be used for in vivo and in vitro optical imaging, since it can not be activated without a UV excitation (254 nm).

Replacing the superparamagnetic single crystals by paramagnetic or by diamagnetic oxide substituted by luminescent Ln cations makes no difference. Down-conversion paramagnetic Gd_2_O_3_:Tb^3+^ [222,223], Dy_2_O_3_:Tb^3+^ [224], and Ho_2_O_3_:Tb^3+^ [225] or diamagnetic Y_2_O_3_:Gd^3+^-Eu^3+^ [226] nanocrystals suffer from the same limitations for in vitro or in vivo imaging.

As an alternative, (Gd, Yb, Tb)PO_4_ nanocrystals were investigated as up-conversion systems. They exhibit ultraviolet, blue, and green up-conversion emissions upon excitation with a 980 nm continuous wave laser diode [227]. They are also efficient T_2_-weighted contrast agents with a r_2_/r_1_ relaxivity ratio (per Gd atom) larger than 2, between 11 and 12 at 20 MHz and 300 K in water for free particles, and around 22 when the particles are coated with Dextran. These results suggest that the lack of water molecules into the Gd inner coordination sphere, capable of exchanging efficiently with the bulk water, leads to inefficient T_1_ relaxation. In other words, the T_2_-relaxation process, which has a strong outer-sphere contribution from field inhomogeneities created by the magnetized particles, that the water protons experience as they diffuse nearby, appears to be more efficient, particularly for the Dextran-coated particles. These results are very promising and open real opportunities for efficient dual MRI and OI applications.

**Figure 22:**
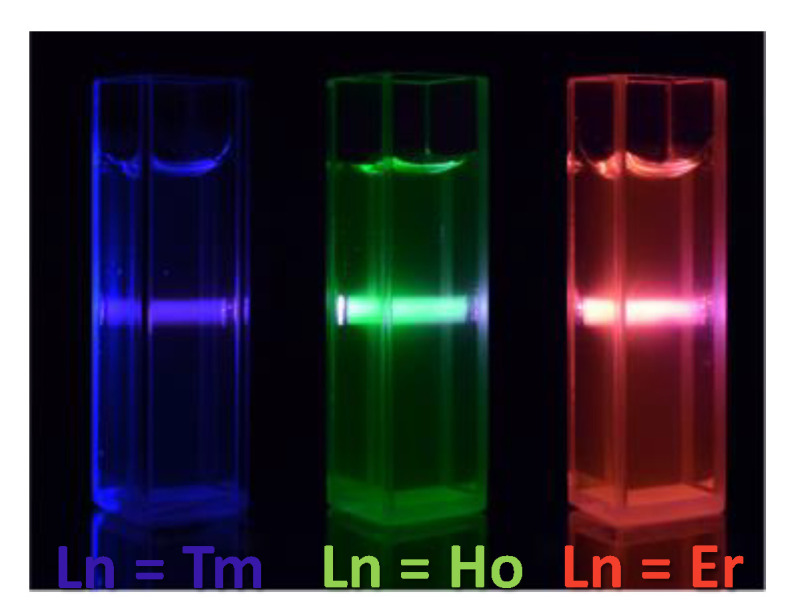
Up-conversion fluorescence images for Gd_2_O_3_:Yb^3+^-Ln^3+^ (Ln = Ho, Er or Tm) colloids under excitation at 980 nm, obtained using a digital camera without any filter. Reproduced from [228], with permission from DOVE Press Publisher, 2016.

Other paramagnetic UCNPs were successfully employed as dual probes. Typically, ultrasmall paramagnetic Gd_2_O_3_ oxide nanocrystals co-doped with luminescent Ln cations like Gd_2_O_3_:Yb^3+^-Er^3+^ [222,228], Gd_2_O_3_:Yb^3+^-Ho^3+^ [228], and Gd_2_O_3_:Yb^3+^-Tm^3+^ [228] proved to be suitable for the desired application. All exhibit strong visible light emission after NIR excitation (Figure 22) and relatively high longitudinal relaxivity, ranging from 13 to 16 mM^−1^.s^−1^ (per Gd atom), in water at 3.0 T and 310 K. The r_1_ value of these contrast agents is proportional to the number of hydration water molecules, which corresponds directly to the number of surface unpaired electrons of Gd^3+^ ions. Compared to the standard Gd-DTPA agent, which has only one Gd^3+^ ion coordinated to only one water molecule, the Gd_2_O_3_:Yb^3+^-Ln^3+^ particles have all their surface Gd^3+^ ions available to bind several water molecules.

Within the same strategy, co-doped diamagnetic nanocrystals like LaF_3_:Yb^3+^-Ho^3+^ were also used. They offer both up-conversion properties and MRI contrast capabilities. Their measured in water r_1_ and r_2_ reached a value of 0.12 and 28.18 mM^−1^.s^−1^ per Ho, at 11.0 T (500 MHz) and 27 °C [229].

#### 4.3.2. Core–Shell Crystalline Hetero-Nanostructures

**Figure 23:**
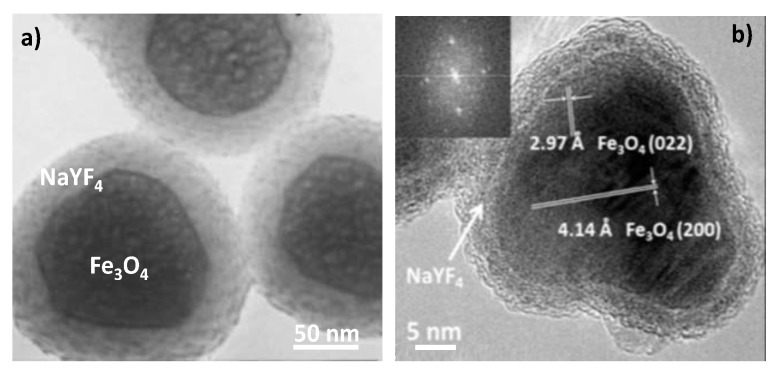
TEM micrographs of representative (**a**) Fe_3_O_4_@NaYF_4_:Yb^3+^-Er^3+^ and (**b**) Fe_3_O_4_@NaYF_4_:Yb^3+^-Tm^3+^ superparamagnetic up-converting core–shell particles. Reproduced from [195] and [230], with permissions from RCS, 2004, and ACS, 2016, respectively.

Structures involving a superparamagnetic iron oxide core coated by an up-conversion-type crystalline shell like Fe_3_O_4_@NaYF_4_:Yb^3+^-Er^3+^ [195], Fe_3_O_4_@NaYF_4_:Yb^3+^-Tm^3+^ [230], Fe_3_O_4_@LaF_3_:Ce^3+^-Tb^3+^ [194] and Co_0.16_Fe_2.84_O_4_@NaYF_4_:Yb^3+^-Er^3+^ [230] have been successfully engineered (Figure 23).

**Figure 24:**
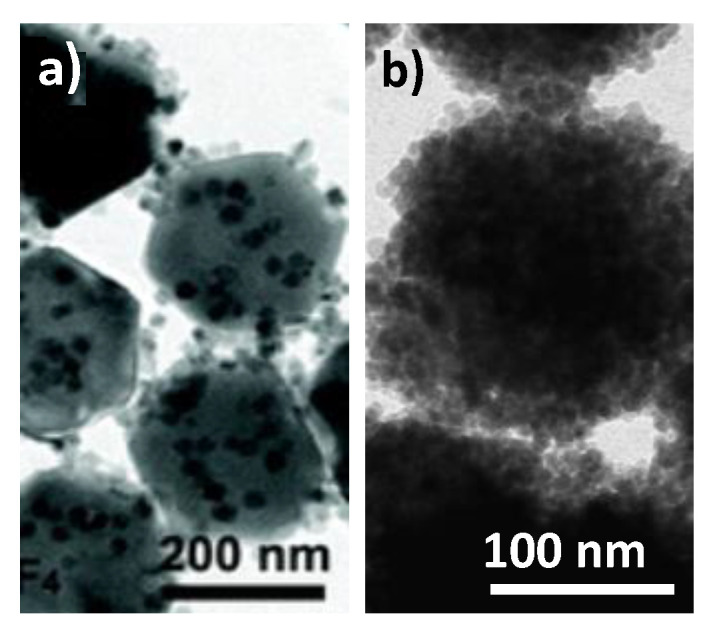
TEM images of (**a**) NaYF_4_:Yb^3+^-Er^3+^@Fe_3_O_4_ and (**b**) NaYF_4_:Eu^3+^@Fe_2_O_3_ particles. Reproduced from [196] and [231], with permissions from John Wiley and Sons, 2014, and MDPI, 2020, respectively.

Conversely, UCNPs surrounded by superparamagnetic iron oxide nanosatellites have been also prepared. Typically, NaYF_4_:Yb^3+^-Er^3+^ particles, about 100 nm in size, were cross-linked to Fe_3_O_4_ particles (less than 10 nm) forming architectures (Figure 24a) exhibiting both significantly intense red emission under NIR excitation (980 nm) and superparamagnetic behavior at room temperature, with a saturation magnetization of about 9 emu.g^−1^ (per mass of powder) [196]. Similar architectures with a richer iron oxide content and then a larger magnetization value have been also prepared using the polyol process (Figure 24b) [231] and successfully evaluated for dual imaging, replacing the luminescent NaYF_4_:Yb^3+^-Er^3+^ UNCP core by a NaYF_4_:YEu^3+^ DCNP one.

**Figure 25:**
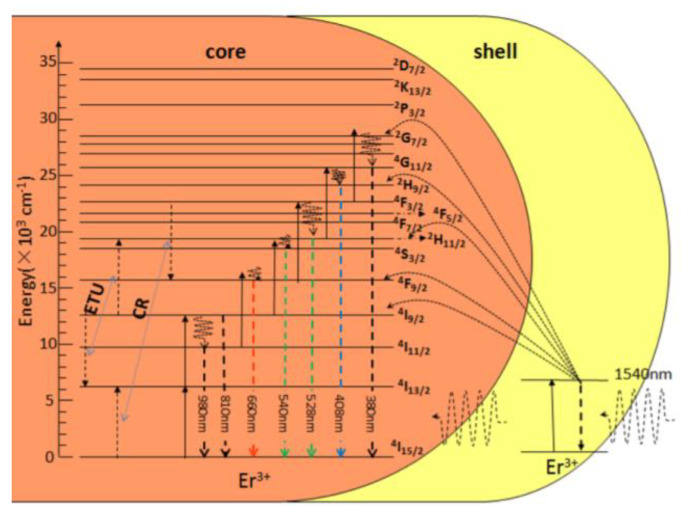
Energy transfers and de-excitations involved in NaGdF_4_:12%Er^3+^@NaGdF_4_:10%Er^3+^ active-core/active-shell NPs under 1540 nm excitation. Reproduced from [232], with permission from Beilstein-Institut Publisher, 2017.

Focusing on UCNP cores, hetero-nanostructures have been also built by growing epitaxially a paramagnetic shell of the same crystallographic type. The first reported system is that of NaYF_4_:Yb^3+^-Er^3+^@NaGdF_4_ nanoconstructs [197,198], which have the advantage of less surface optical quenching thanks to the spatial isolation of the luminescent core from its environments. Similar architectures were prepared based on singly Er-doped paramagnetic NaGdF_4_:Er^3+^@NaGdF_4_:Er^3+^ core-shell particles [232]. By adjusting the Er concentration in the shell and in the core, for example, 10% in the core and 12 at % in the shell, the active shell may play the role of a sensitizer for the luminescence of the Ln^3+^ cations in the core (Figure 25). Green and red emissions were thus obtained under a 1540 nm excitation thanks to this process [232]. Of course, the availability of Gd^3+^ ions at the probe surface makes the engineered core–shell particles valuable for inner-sphere magnetic interaction with water molecules and, therefore, efficient positive MRI contrasting.

## 5. Conclusions

After introducing the basic principles of MRI and OI and describing the various probes used to enhance image contrast in one case and provide a light source in the other case, we have reviewed the different nanometric magnetic and luminescent architectures that can be used simultaneously for bimodal imaging, focusing on the lanthanide luminescence phenomenon within single objects. We have classified the corresponding bimodal probes into three families, the all-molecular ones, the hybrids, and the all-inorganic ones. Each of them has advantages and drawbacks for in vivo dual imaging but, in all the cases, their optical properties remain excellent, particularly when they involve the up-conversion feature. Their fluorescence intensity, brightness, photostability, the width of excitation wavelength window and narrow emission window, etc. are often good, making them very efficient biomarkers. This is particularly true when they can be excited by NIR light to avoid tissue scattering and limit luminescence quenching in an aqueous environment. The engineered probes may also be efficient MRI contrast agents, taking advantage of high molecular weight and then a long tumbling time, a high exposed surface and therefore a large number of interacting water molecules, a high concentration of paramagnetic cations in a small volume which gives high field inhomogeneity, and other factors reinforcing the contrast. Finally, dual imaging contrast agents could provide diagnostic information at the early stages of certain diseases and avoid invasive procedures, since they overcome the low sensitivity of MRI and the low detection limit of OI.

## Figures and Tables

**Table 1:** Longitudinal relaxivities r_1_ of different Gd-based CAs

	Number Gd^3+^/CA	CA weight kD	r_1_/CA mM^−1^.s^−1^	r_1_/Gd^3+^ mM^−1^.s^−1^	T °C	*f*_RF_ MHz	Ref.
Gd-DTPA	1	0.6	3.7	3.7	37	20	[2]
Gd-DOTA	1	*−*	4,2	4.2	25	20	[2]
Dextran-Gd-DTPA	15	75	57	11.0	37	20	[32]
Polylysine-Gd-DTPA	60	48.7	850	13.1	39	20	[35]
Albumin-Gd-DTPA	90	90	420	14.0	25	10	[45]
6-dendrimer-Gd-DTPA	170	139	5800	34.0	37	*−*	[46]
Gd-NTCs	*−*	0.044–0.049	173–164	40	60	*−*	[47]
Gd-DOTA-Apoferritin	6	*−*	3.9	25	64	*−*	[43]
Gd-Me2DO2A-Apoferritin	36	*−*	35.9	25	64	*−*	[43]

**Table 2:** Longitudinal relaxivity r_1_ for different paramagnetic inorganic NPs under different MRI operating conditions

	Size nm	r_1_/M^n+^ mM^−1^.s^−1^	T °C	B_0_ T	Ref.
MnO spheres	7 20	0.37 0.13	25	3.0	[49]
MnO hollow spheres	20	1.15	25	1.5	[56]
Mn_3_O_4_ spheres	9.8	1.31	25	3.0	[48]
Mn_3_O_4_ platelets	10	2.06	25	3.0	[48]
Gd_2_O_3_ particles	5	9.2	21–23	1.5	[52]

**Table 3:** Main characteristics of iron oxide-based negative CAs. Note, the reported r_2_ and r_2_/r_1_ values were expressed per mM of iron atoms.

	Composition	Size nm	r_2_ mM^−1^.s^−1^	r_2_/r_1_	T °C	B_0_ T	Ref.
SPIO	Fe_2_O_3_ NPs in Dextran	50–100	160	4.0	37	0.47	[72]
	Fe_2_O_3_ NPs in carboxylate Dextran	30–50	190	7.9	37	0.47	
USPIO	Fe_2_O_3_ NPs in Dextran	17–20	53	2.2	37	0.47	
MION	Fe_2_O_3_ NPs in Dextran	18–24	35	2.2	37	0.47	

**Table 4:** Main characteristics of non-iron oxide negative CAs

	Composition	Size nm	r_2_ mM^−1^.s^−1^	T °C	B_0_ T	Ref
Manganite	57 nm sized La_0.75_Sr_0.25_MnO_3_ NPs coated with a silica layer of 80–100 nm in thickness	150	580 ^a^ 540 ^a^ 520 ^a^	20 20 20	0.5 1.5 3.0	[62]
Ferrite	4 nm sized CoFe_2_O_4_ NPs in carboxylate PEG	30	185 ^b^	25	1.5	[73]
	8 nm sized MnFe_2_O_4_ NPs in PEG-PCL	80	66 ^b^	25	1.5	
Fe	Less than 10 nm sized Fe NPs coated by PEG	10	129 ^c^	25	1.5	[74]
EFNPs	Fe@Ni_x_Fe_3-x_O_4_ NPs coated by PEG	15	9.96 ^d^	25	2.4	[70]

Expressed per mM of (^a^) manganese atoms, (^b^) paramagnetic cations, (^c^) iron atoms, or (^d^) particles.

**Table 5:** Main spectroscopic characteristics of luminescent semiconducting nanocrystals for optical fluorescence imaging. ε is the molar extinction coefficient associated with the main absorption, FWHM the width at half height of the emission band and Ф_f_ the quantum yield.

QD	λ_absorption_ (nm)	λ_emission_ (nm)	FWHM (nm)	ε (M^−1^ cm^−1^)	Ф_f_ (%)	Ref.
CdS	350–470	370–500	~30	1.0 × 10^5^ (at 350 nm) 9.5 × 10^5^ (at 450 nm)	≤60 ^a^	[118]
CdSe	450–640	470–660	~30	1.0 × 10^5^ (at 500 nm) 7.0 × 10^5^ (at 630 nm)	65 ^a^ 8 ^a^	[119]
CdTe	500–700	520–750	35–45	1.3 × 10^5^ (at 570) 6.0 × 10^5^ (at 700 nm)	30 ^a^ 75 ^a^	[120,121]
PbS	800–3000	>900	80–90	−	26 ^b^ 70 ^c^	[122,123,124]
PbSe	900–4000	>1000	80–90	1.23 × 10^5^	45 ^d^	[125,126]
InP	550–650	620–720	50–90	−	10–60	[127,128]

(^a^) in CH_3_OH, (^b^) in HEPES, (^c^) in C_6_H_14_, and (^d^) in CHCl_3_.

**Table 6:** Optical characteristics of representative UCNPs

Host Lattice	Sensitizer	Activator	Major Emission (nm)	Ref.
NaYF_4_	Yb	Er	518, 537 and 652	[169]
		Er	540 and 660	[168]
		Er	521, 539 and 651	[174]
		Tm	450, 475 and 647	[168]
		Ho	540	[175]
		Ho	542 and 645, 658	[176]
LaF_3_	Yb	Er	520, 545 and 659	[177]
		Tm	475	[177]
		Ho	542 and 645, 658	[177]
CaF_2_	Yb	Er	524 and 654	[178]
Y_2_O_3_	Yb	Er	550 and 660	[161]
		Ho	543 and 665	[179]
LuPO_4_	Yb	Tm	475 and 649	[166]
